# Association between sleep duration and three obesity indicators among middle-aged and elderly adults: findings from the NHANES 2005–2014

**DOI:** 10.3389/fnut.2024.1464851

**Published:** 2024-11-13

**Authors:** Mengjin Jiang, Binyao Shou, Lihua Shi, Min He

**Affiliations:** ^1^Department of Pharmacy, The First People’s Hospital of Xiaoshan District, Hangzhou, China; ^2^Department of Pharmacy, The Second People’s Hospital of Xiaoshan District, Hangzhou, China

**Keywords:** body mass index (BMI), waist circumference (WC), waist-to-height ratio (WHtR), sleep duration, obesity, middle-aged and elderly people, NHANES

## Abstract

**Objective:**

Obesity has emerged as a worldwide problem. In recent years, it has been verified that there is an association between sleep duration and obesity indicators. This provides a new approach to control obesity. In this study, the relationship between duration of sleep and body mass index (BMI), waist circumference (WC), and waist-to-height ratio (WHtR) among Americans *≥*45 years old was investigated.

**Methods:**

Data was collected from the National Health and Nutrition Examination Survey (NHANES) between 2005 and 2014. The link between sleep duration and obesity indicators was analysed using multiple regression models and weighted smoothed curve fitting. Subgroup analysis was conducted to assess the consistency of the connection between sleep duration and obesity indicators across various groups.

**Results:**

This study involved 7,118 males and 7,265 females, with an average age of 62.09. After total adjustment, sleep duration was negatively correlated with BMI (β = −0.19, 95%CI: −0.26, −0.12), WC (β = −0.36, 95%CI: −0.53, −0.19) and WHtR (β = −0.27, 95%CI: −0.38, −0.17). Subgroup analyses revealed more significant negative associations between sleep duration and BMI, WC, and WHtR among non-Hispanic White participants, and those without diabetes and hypertension.

**Conclusion:**

Sleep duration was significantly negatively associated with BMI, WC, and WHtR, suggesting that longer sleep duration may contribute to lower obesity indicators in middle-aged and elderly Americans. Subgroup analysis showed that their negative correlation differed between races, diabetes, or hypertension status. However, additional prospective studies are required to validate these findings and investigate potential causal relationships.

## Introduction

Over the past few decades, obesity has emerged as a severe health problem in the world since it can increase the incidence of ailments such as cardiovascular disease, diabetes, and cancer, especially among middle-aged and elderly age populations (1). The World Health Organization (WHO) characterizes obesity as the excessive accumulation of adipose tissue beyond normal physiological requirements (2). The Centers for Disease Control and Prevention’s Prevalence of Obesity in Adults 2023 map (3)revealed that middle-aged adults were nearly twice as likely to be obese compared to young adults across all states. Obesity rates were lowest among 18- to 24-year-olds at 19.5% and highest among 45- to 54-year-olds at 39.2%. Slow metabolism, decreased exercise, unbalanced diet and energy intake, as well as life pressure, have led to a greater risk of obesity for middle-aged and elderly people (4). Therefore, it is essential to take suitable actions to prevent and manage obesity. Obesity is characterized by a prolonged imbalance between caloric intake and energy expenditure, typically indicated by a BMI of 30 or higher (5). Adopting habits that decrease energy intake while boosting calorie expenditure might help slow down the advancement of obesity, including following a proper diet and engaging in physical activity (6). In addition, sleep may be another important factor (7).

Sufficient sleep is favorable for regulating the immune system and human metabolism (8). On the contrary, insufficient sleep may result in metabolic disturbances (9). Generally, 7–9 h is deemed as the suitable sleep time for overall physical and mental (10). However, over one-third of American adults get less than 7 h of sleep (11). A similar trend of shorter sleep duration has been observed in some Western countries over the past decades (7). Thus, a comprehensive evaluation of the correlation between sleep duration and obesity is crucial for exploring weight management (12, 13).

Numerous previous researches have confirmed the correlation between the duration of sleep and obesity (14–30). Nevertheless, these articles mainly found a negative relationship between short sleep time and obesity, and the results of long sleep time were unclear. In addition, the amount of sleep required varies at different ages. This study sought to elucidate further the relationship between sleep duration and various anthropometric indices of obesity in middle-aged and elderly Americans, ≥45 years old, using the data from the National Health and Nutrition Examination Surveys (NHANES) 2005–2014. Currently, the indices typically employed for clinical evaluation of obesity include waist circumference (WC), waist-to-hip ratio (WHR), waist-to-height ratio (WHtR), and body mass index (BMI) (31–33). Due to some limitations of the National Health and Nutrition Examination Survey (NHANES) surveys analyzed in this study (missing data on the hip circumference in the cited surveys), we selected BMI, WC, and WHtR as measures of obesity in this paper.

## Methods

### Targeted population

**Figure 1:**
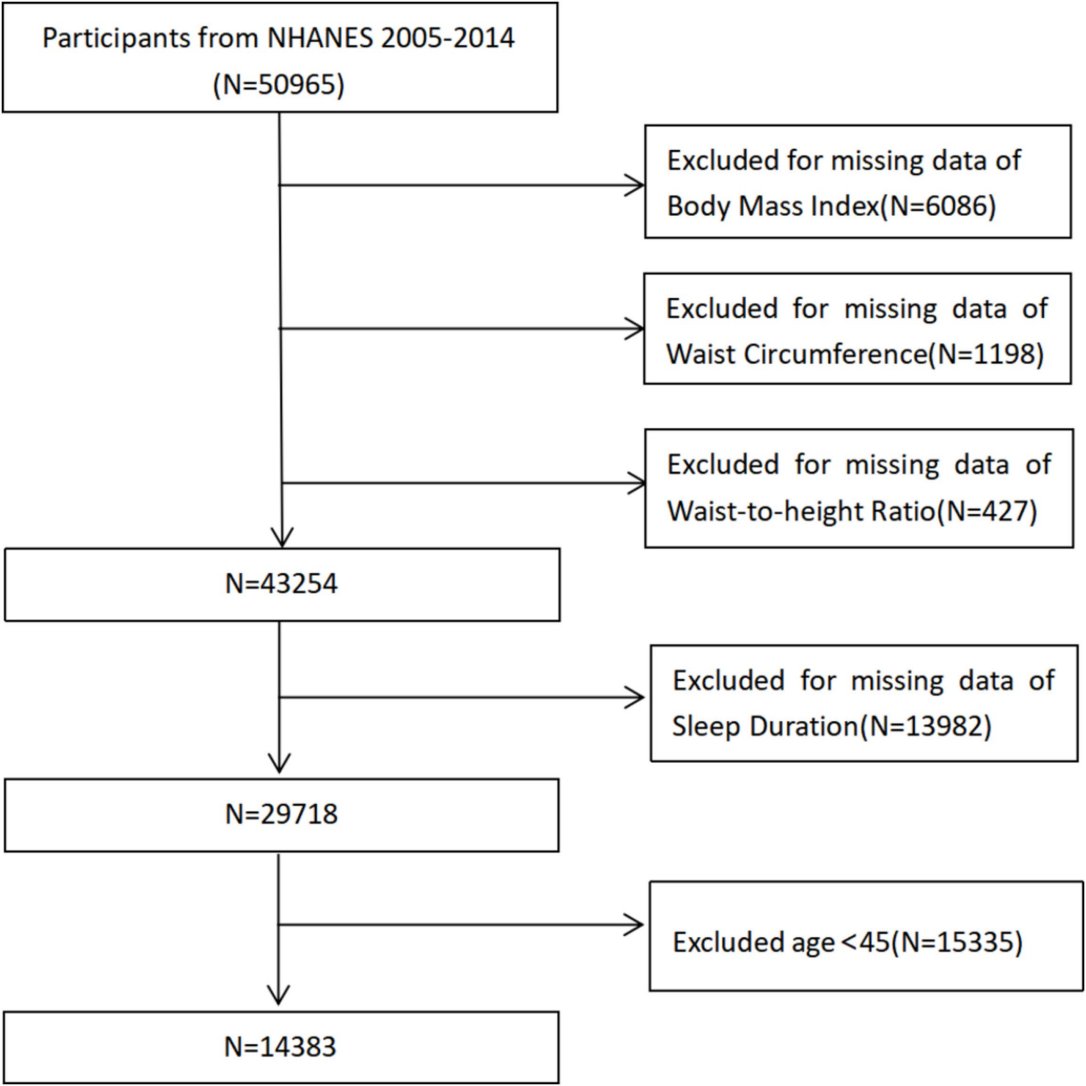
Participant selection flowchart. NHANES, National Health and Nutrition Examination Survey.

The National Health and Nutrition Examination Survey (NHANES) refers to a sizeable cross-sectional study to evaluate youths and adults’ health and nutritional condition throughout the U.S. (34). The survey annually assesses a nationally representative sample comprising approximately 5,000 individuals. These participants are distributed across various counties nationwide, with 15 specific counties being visited each year (35). The National Center for Health Statistics Ethics Review Board granted approval for the NHANES protocols, ensuring that all participants provided written informed permission. And that the data could be made publicly available for further analysis. As per the regulation of the National Institutes of Health (NIH), studies that use de-identified data and do not include direct interaction with participants are not classified as human subjects research. They do not require review by the institutional review board. Data from the NHANES 2005–2014 surveys were collected, excluding participants lacking BMI (*n* = 6,086), WC (*n* = 1,198), WHtR (*n* = 427) or sleep duration data (*n* = 13,982). Additionally, subjects younger than 45 (*n* = 15,335) were removed. Finally, 14,383 individuals were included in this study (Figure 1).

### Data and variables

Three obesity indicators and sleep duration were set as the dependent and targeted independent variables, respectively. According to international standards, BMI was calculated as weight divided by height squared, while WHtR was calculated as WC divided by height (36), and in this study was expressed as a percentage, due to more observable effects in regression analyses. NHANES database provides accurate measurements of patients’ BMI, WC and WHtR by professionally tested healthcare professionals. Participants provided self-reported information on their sleep duration. This question determined the duration of sleep time: “How much sleep do you usually get at night on weekdays or workdays?” Based on the expert advice of the American Academy of Sleep Medicine and the Sleep Research Society (37, 38), it was categorized into three categories: short sleep time (<7 h/day), regular sleep time (7–9 h/day) and long sleep time (>9 h/day).

Based on similar studies (12, 28, 39), various covariates that may influence the outcome were considered, including age, gender, race, education, marital status, family PIR (income to poverty ratio), alcohol status, smoke status, moderate work activity, moderate recreational activities, diabetes, and hypertension status. Comprehensive explanations for all variables can be found on the official page of the NHANES website.^1^

### Statistical analysis

Statistical analyses were carried out utilising R (version 4.1.3) and Empower Stats (version 2.0). The Kolmongorow–Smirnow test was used to test if the numeric variables were normally distributed or not. Parametric numeric data were presented as mean (SD, standard deviation), non-parametric numeric data were presented as median (IQR), and categorical variables were presented as a number (percentage). Differences between subgroups of subjects classified by sleep duration were evaluated using an analysis of variance (ANOVA) with Bonferroni *post hoc* test (parametric numeric data), Kruskal-Wallis test with *post hoc* Dunn’s test (non-parametric numeric data), or chi-square test (categorical data). The linear association between sleep duration and obesity indicators were examined using multiple regression models, and their relationship was further analysed by a weighted smoothed curve fitting method. Subgroup analyses were adopted to determine whether the associations of sleep duration with BMI, WC, and WHtR were stable in different populations (classified by gender, race, diabetes, or hypertension status). For covariates with more missing data, median interpolation was carried out for continuous variables, and logistic regression interpolation was carried out for categorical variables. For other covariates with less missing data, the subjects with missing values were removed from the analyses.

## Results

### Baseline characteristics

This study involved a total of 14,383 participants, consisting of 7,118 males and 7,265 females, with an average age of 62.09 ± 10.97 years. Their average BMI, WC, WHtR and sleep duration were 29.20 ± 6.40 kg/m^2^, 101.28 ± 15.12 cm, 0.61 ± 0.09, and 6.85 ± 1.48 h/day, respectively. The group with the shortest sleep duration had the highest obesity rates (BMI ≥ 30). Those who slept more than 9 h were older, had fewer work and leisure activities, were more sedentary, and had higher rates of diabetes and hypertension compared to those who slept less than 9 h. Meanwhile, people who slept 7–9 h had the highest levels of education, the lowest rates of smoking, diabetes and hypertension, and the most moderate work and leisure activities (Table 1).

**Table 1:** Characteristics of participants grouped by short, normal, and long sleep duration.

	Total (*n* = 14,383)	Sleep duration (h/day)			*p-*Value	<7 h vs. 7–9 h	<7 h vs. >9 h	7–9 h vs. >9 h
		<7 h (*n* = 5,648)	7–9 h (*n* = 8,317)	>9 h (*n* = 418)				
Age (years)	61.00 (53.00–71.00)	60.00 (52.00–68.00)	62.00 (53.00–72.00)	69.00 (59.00–80.00)	<0.001	<0.001	<0.001	<0.001
Gender, *n* (%)					0.658	0.966	0.632	0.680
Male	7,118 (49.49)	2,806 (49.68)	4,114 (49.46)	198 (47.37)				
Female	7,265 (50.51)	2,842 (50.32)	4,203 (50.54)	220 (52.63)				
Race, *n* (%)					<0.001	<0.001	0.520	0.308
Mexican American	1,939 (13.48)	753 (13.33)	1,143 (13.74)	43 (10.29)				
Other Hispanic	1,270 (8.83)	548 (9.70)	688 (8.27)	34 (8.13)				
Non-Hispanic White	6,956 (48.36)	2,244 (39.73)	4,483 (53.90)	229 (54.78)				
Non-Hispanic Black	3,126 (21.73)	1,629 (28.84)	1,407 (16.92)	90 (21.53)				
Other race	1,092 (7.59)	474 (8.39)	596 (7.17)	22 (5.26)				
Education, *n* (%)					<0.001	<0.001	<0.001	<0.001
<High school	4,221 (29.35)	1714 (30.35)	2,325 (27.96)	182 (43.54)				
High school	3,354 (23.32)	1,373 (24.31)	1,877 (22.57)	104 (24.88)				
>High school	6,808 (47.33)	2,561 (45.34)	4,115 (49.47)	132 (31.58)				
Marital status, *n* (%)					<0.001	<0.001	0.699	0.004
Married or living with partner	8,772 (60.99)	3,258 (57.68)	5,315 (63.90)	199 (47.6)				
Widowed	1,938 (13.47)	734 (13.00)	1,099 (13.21)	105 (25.12)				
Divorced or separated	2,606 (18.12)	1,186 (20.99)	1,351 (16.25)	69 (16.51)				
Never married	1,067 (7.42)	470 (8.33)	552 (6.64)	45 (10.77)				
Diabetes status, *n* (%)					<0.001	0.005	0.004	<0.001
Yes	2,623 (18.24)	1,107 (19.60)	1,412 (16.98)	104 (24.88)				
No	11,308 (78.62)	4,352 (77.05)	6,649 (79.94)	307 (73.44)				
Borderline	452 (3.14)	189 (3.35)	256 (3.08)	7 (1.67)				
Family PIR	2.25 (1.26–4.12)	2.25 (1.18–3.87)	2.25 (1.34–4.38)	1.71 (1.01–2.57)	<0.001	<0.001	<0.001	<0.001
Had at least 12 alcohol drink a year, *n* (%)	10,196 (70.89)	3,954 (70.01)	5,959 (71.65)	283 (67.70)	0.039	0.091	0.576	0.193
Current smoking, *n* (%)	2,618 (18.20)	1,209 (21.4)	1,311 (15.77)	98 (23.44)	<0.001	<0.001	0.189	<0.001
Moderate work activity, *n* (%)	3,742 (26.02)	1,554 (27.51)	2,126 (25.56)	62 (14.83)	<0.001	0.026	<0.001	<0.001
Moderate recreational activities, *n* (%)	4,620 (32.12)	1,647 (29.16)	2,884 (34.68)	89 (21.29)	<0.001	<0.001	0.002	<0.001
Sedentary activity (min/day)	300.00 (240.00–480.00)	300.00 (240.00–480.00)	300.00 (240.00–480.00)	360.00 (300.00–480.00)	<0.001	0.033	<0.001	<0.001
Hypertension, *n* (%)	7,320 (50.89)	2,998 (53.08)	4,058 (48.79)	264 (63.16)	<0.001	<0.001	<0.001	<0.001
WC (cm)	100.20 (91.00–110.10)	100.90 (91.30–111.50)	99.90 (90.80–109.30)	100.15 (90.83–110.77)	<0.001	<0.001	1.000	1.000
WHtR (%)	60.24 (54.67–66.35)	60.57 (54.98–67.09)	59.94 (54.52–65.78)	60.99 (55.01–67.30)	<0.001	<0.001	1.000	0.201
BMI (kg/m^2^)	28.26 (24.80–32.42)	28.75 (25.10–33.20)	28.00 (24.70–32.00)	27.51 (24.10–32.38)	<0.001	<0.001	0.002	0.816
BMI (kg/m^2^), *n* (%)					<0.001	<0.001	0.002	0.816
	208 (1.45)	86 (1.52)	108 (1.30)	14 (3.35)				
18.5–25	3,544 (24.64)	1,287 (22.79)	2,143 (25.77)	114 (27.27)				
25–30	5,115 (35.56)	1,923 (34.05)	3,048 (36.65)	144 (34.45)				
≥ 30	5,516 (38.35)	2,352 (41.64)	3,018 (36.29)	146 (34.93)				

PIR, poverty income ratio; WC, waist circumference; WHtR, waist-to-height ratio; BMI, body mass index.

### The association between sleep duration and obesity indicators (BMI, WC, WHtR)

The outcomes of the multivariate logistic regression analysis with the three models are displayed in Tables 2–4. Sleep duration was negatively associated with BMI in all three models. In the multivariate model (Model 3), after full adjustment, a one-hour increase in sleep duration was associated with a 0.19 kg/m^2^ decrease in BMI (β = −0.19, 95%CI: −0.26, −0.12). The duration of sleep was divided into three groups for detailed analysis, converting it from a continuous variable to a categorical variable. Compared to the control group (<7 h), the group with the maximum sleep duration (>9 h) had a decrease in BMI of 1.28 kg/m^2^ (β = −1.28, 95%CI: −1.93, −0.62), which was significantly higher than the decrease of 0.51 kg/m^2^ (β = −0.51, 95%CI: −0.72, −0.31) in the 7–9 h group (Table 2).

**Table 2:** Association of sleep duration and BMI.

Exposure	Model 1 β (95%CI)	Model 2 β (95%CI)	Model 3 β (95%CI)
Sleep duration	−0.32 (−0.40, −0.24) *p* < 0.0001	−0.26 (−0.33, −0.18) *p* < 0.0001	−0.19 (−0.26, −0.12) *p* < 0.0001
Sleep duration classification			
<7 h	Reference	Reference	Reference
7–9 h	−0.97 (−1.19, −0.75) *p* < 0.0001	−0.82 (−1.04, −0.60) *p* < 0.0001	−0.51 (−0.72, −0.31) *p* < 0.0001
>9 h	−1.24 (−1.96, −0.53) *p* = 0.0006	−0.95 (−1.66, −0.24) *p* = 0.0088	−1.28 (−1.93, −0.62) *p* = 0.0001
*p* for trend	<0.001	<0.001	<0.001

Model 1: Not adjusted for covariates; Model 2: Adjusted for age, gender, and race; Model 3: Adjusted for age, gender, race, education, marital status, family PIR, alcohol status, smoke status, moderate work activity, moderate recreational activities, diabetes, hypertension. PIR, poverty income ratio; BMI, body mass index.

**Table 3:** Association of sleep duration and WC.

Exposure	Model 1 β (95%CI)	Model 2 β (95%CI)	Model 3 β (95%CI)
Sleep duration	−0.51 (−0.70, −0.33) *p* < 0.0001	−0.54 (−0.73, −0.36) *p* < 0.0001	−0.36 (−0.53, −0.19) *p* < 0.0001
Sleep duration classification			
<7 h	Reference	Reference	Reference
7–9 h	−1.62 (−2.14, −1.09) *p* < 0.0001	−1.67 (−2.19, −1.15) *p* < 0.0001	−0.80 (−1.28, −0.31) *p* = 0.0012
>9 h	−1.81 (−3.52, −0.09) *p* = 0.0390	−1.85 (−3.52, −0.17) *p* = 0.0308	−2.78 (−4.33, −1.24) *p* = 0.0004
*P* for trend	<0.001	<0.001	<0.001

Model 1: Not adjusted for covariates; Model 2: Adjusted for age, gender, and race; Model 3: Adjusted for age, gender, race, education, marital status, family PIR, alcohol status, smoke status, moderate work activity, moderate recreational activities, diabetes, hypertension. PIR, poverty income ratio; WC, waist circumference.

**Table 4:** Association of sleep duration and WHtR.

Exposure	Model 1 β (95%CI)	Model 2 β (95%CI)	Model 3 β (95%CI)
Sleep duration	−0.29 (−0.40, −0.18) *p* < 0.0001	−0.40 (−0.51, −0.29) *p* < 0.0001	−0.27 (−0.38, −0.17) *p* < 0.0001
Sleep duration classification			
<7 h	Reference	Reference	Reference
7–9 h	−1.08 (−1.39, −0.77) *p* < 0.0001	−1.23 (−1.54, −0.92) *p* < 0.0001	−0.63 (−0.92, −0.34) *p* < 0.0001
>9 h	−0.34 (−1.35, 0.68) *p* = 0.5159	−1.15 (−2.16, −0.14) *p* = 0.0251	−1.88 (−2.80, −0.96) *p* < 0.0001
*p* for trend	<0.001	<0.001	<0.001

Model 1: Not adjusted for covariates; Model 2: Adjusted for age, gender, and race; Model 3: Adjusted for age, gender, race, education, marital status, family PIR, alcohol status, smoke status, moderate work activity, moderate recreational activities, diabetes, hypertension. PIR, poverty income ratio; WHtR, waist-to-height ratio.

There was also a significant negative association between sleep duration and WC (β = −0.36, 95%CI: −0.53, −0.19). After full adjustment, a one-hour increase in sleep duration was associated with a 0.36 cm decrease in WC. Similar to the BMI, the most extended sleep duration group (>9 h) had a significantly higher decline (β = −2.78, 95%CI: −4.33, −1.24) in WC compared to the control group (<7 h) than the 7–9 h group (β = −0.80, 95%CI: −1.28, −0.31) (Table 3).

There was also a significant negative association between sleep duration and WHtR (β = −0.27, 95%CI: −0.38, −0.17). After full adjustment, a one-hour increase in sleep duration was associated with a 0.27% decrease in WHtR expressed as a percentage. The >9 h group had a significantly higher decline (β = −1.88, 95%CI: −2.80, −0.96) in WHtR compared to the <7 h group than the 7–9 h group (β = −0.63, 95%CI: −0.92, −0.34) (Table 4).

**Figure 2:**
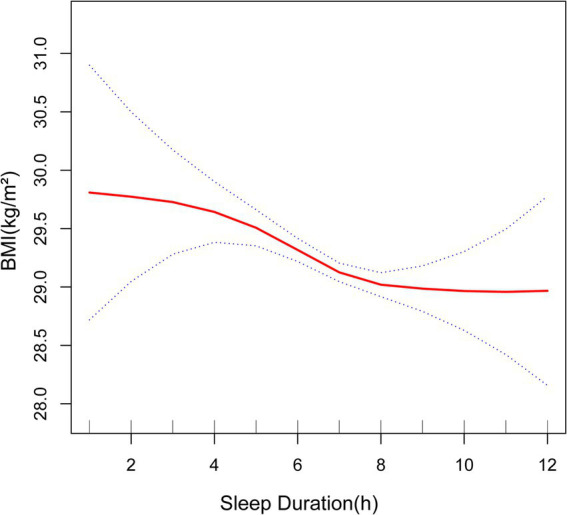
Association between sleep duration and BMI. The solid red line represents the smooth curve fit between variables. Blue bands represent the 95% of confidence interval from the fit. BMI, body mass index.

**Figure 3:**
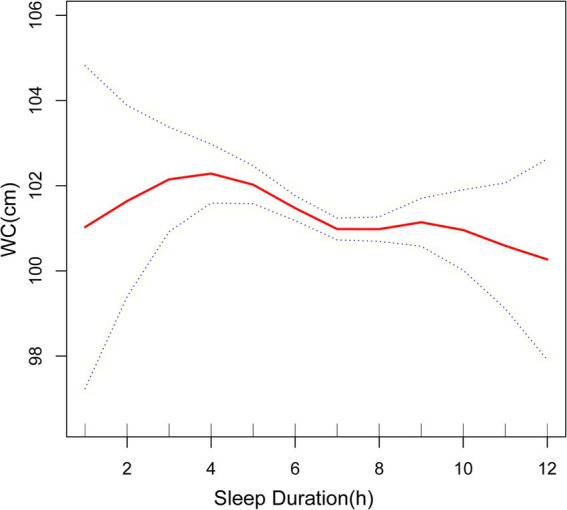
Association between sleep duration and WC. The solid red line represents the smooth curve fit between variables. Blue bands represent the 95% of confidence interval from the fit. WC, waist circumference.

**Figure 4:**
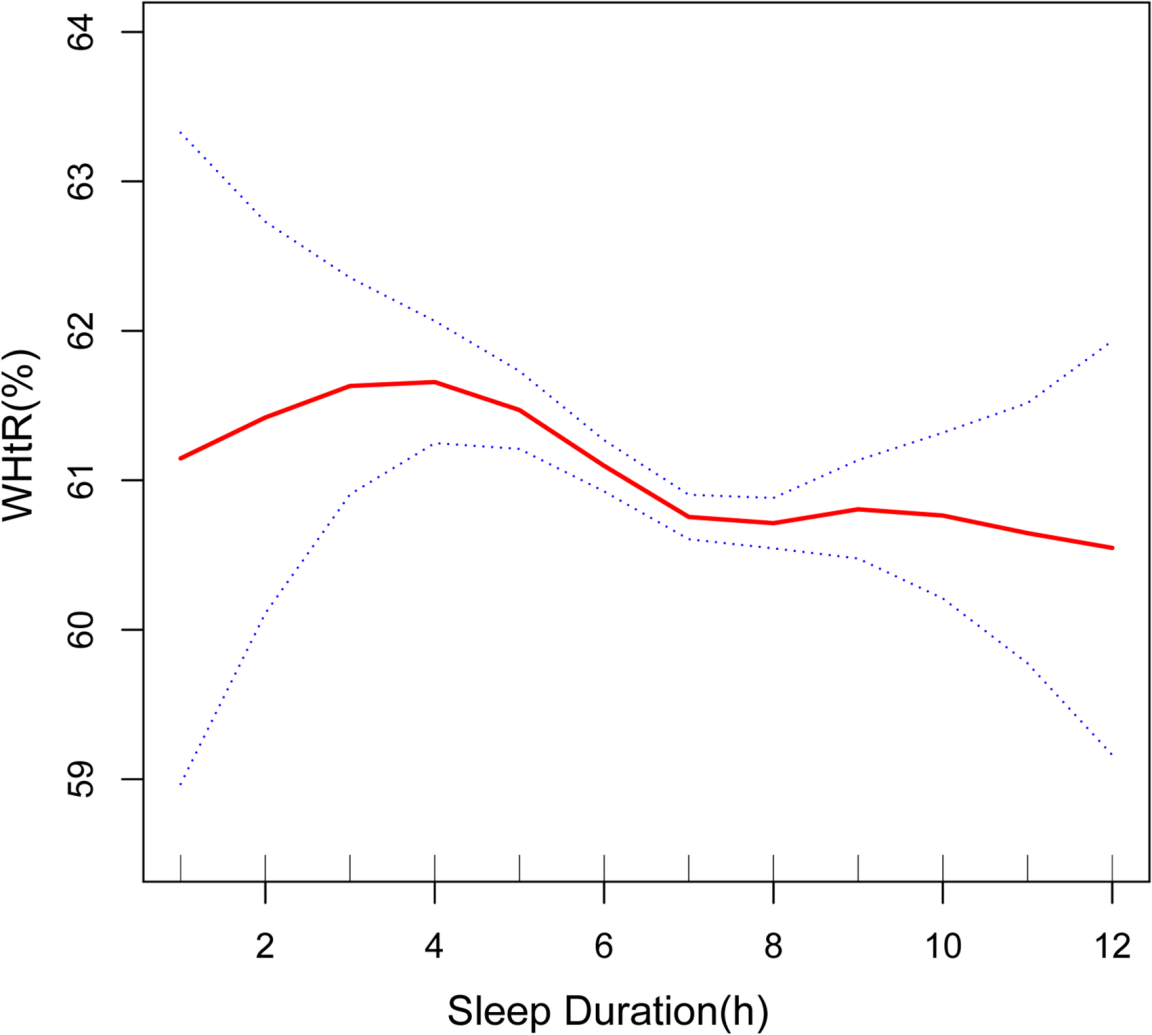
Association between sleep duration and WHtR. The solid red line represents the smooth curve fit between variables. Blue bands represent the 95% of confidence interval from the fit. WHtR, waist-to-height ratio.

Furthermore, the smoothed curve further verified the negative correlation between sleep duration and BMI, WC and WHtR (Figures 2–4). The curve seemed to show a cut-off point at 7 h or 8 h, before which the association was stronger (with a higher slope), after which the effect was less pronounced (with a lower slope), but the *p*-value was nonsignificant.

### Subgroup analyses

The subgroup analyses revealed that sleep duration was negatively associated with BMI, WC, and WHtR in both genders, but particularly among Non-Hispanic White, non-diabetic, and non-hypertensive subjects (Tables 5–7).

**Table 5:** Subgroup analysis of the association between sleep duration and BMI.

Subgroup	OR (95%CI)	*P* for interaction
Gender		0.9279
Male	−0.19 (−0.30, −0.08) *p* = 0.0005	
Female	−0.20 (−0.29, −0.10) *p* < 0.0001	
Race		0.0371
Mexican American	−0.04 (−0.34, 0.27) *p* = 0.8059	
Other Hispanic	0.08 (−0.25, 0.41) *p* = 0.6452	
Non-Hispanic White	−0.25 (−0.33, −0.16) *p* < 0.0001	
Non-Hispanic Black	−0.16 (−0.35, 0.03) *p* = 0.0975	
Other Race	0.14 (−0.15, 0.44) *p* = 0.3457	
Diabetes status		0.7323
Yes	−0.12 (−0.29, 0.06) *p* = 0.0979	
No	−0.18 (−0.26, −0.10) *p* < 0.0001	
Borderline	−0.26 (−0.67, 0.15) *p* = 0.1894	
Hypertension status		0.0383
Yes	−0.10 (−0.20, 0.00) *p* = 0.0521	
No	−0.25 (−0.35, −0.15) *p* < 0.0001	

BMI, body mass index.

**Table 6:** Subgroup analysis of the association between sleep duration and WC.

Subgroup	OR (95%CI)	*P* for interaction
Gender		0.2178
Male	−0.44 (−0.69, −0.19) *p* = 0.0005	
Female	−0.23 (−0.46, −0.00) *p* = 0.0469	
Race		
Mexican American	0.08 (−0.63, 0.79) *p* = 0.8270	0.0250
Other Hispanic	0.40 (−0.37, 1.18) *p* = 0.3053	
Non-Hispanic White	−0.50 (−0.70, −0.29) *p* < 0.0001	
Non-Hispanic Black	−0.30 (−0.75, 0.15) *p* = 0.1888	
Other Race	0.35 (−0.34, 1.04) *p* = 0.3147	
Diabetes status		0.9606
Yes	−0.34 (−0.74, 0.06) *p* = 0.0963	
No	−0.34 (−0.53, −0.15) *p* = 0.0005	
Borderline	−0.20 (−1.17, 0.77) *p* = 0.6901	
Hypertension status		0.1988
Yes	−0.25 (−0.48, −0.01) *p* = 0.0385	
No	−0.47 (−0.71, −0.22) *p* = 0.0002	

WC, waist circumference.

**Table 7:** Subgroup analysis of the association between sleep duration and WHtR.

Subgroup	OR (95%CI)	*P* for interaction
Gender		0.5963
Male	−0.29 (−0.44, −0.14) *p* = 0.0001	
Female	−0.24 (−0.37, −0.10) *p* = 0.0007	
Race		0.0107
Mexican American	−0.01 (−0.43, 0.42) *p* = 0.9711	
Other Hispanic	0.13 (−0.34, 0.59) *p* = 0.5926	
Non-Hispanic White	−0.37 (−0.49, −0.25) *p* < 0.0001	
Non-Hispanic Black	−0.15 (−0.42, 0.12) *p* = 0.2806	
Other Race	0.21 (−0.20, 0.63) *p* = 0.3128	
Diabetes status		0.9086
Yes	−0.21 (−0.45, 0.03) *p* = 0.0822	
No	−0.27 (−0.39, −0.16) *p* < 0.0001	
Borderline	−0.25 (−0.83, 0.34) *p* = 0.4068	
Hypertension status		0.0498
Yes	−0.13 (−0.27, 0.01) *p* = 0.0650	
No	−0.33 (−0.48, −0.19) *p* < 0.0001	

WHtR, waist-to-height ratio.

## Discussion

The purpose of this research was to assess the correlation between sleep duration and three obesity indicators among middle-aged and elderly individuals in the United States. Our cross-sectional study included 14,383 middle-aged and elderly Americans from NHANES. After multiple data analyses, it was concluded that sleep duration was significantly negatively correlated with BMI, WC and WHtR in this population.

Examining three obesity indicators and sleep duration enhanced our conclusions and their applicability. BMI is commonly used for assessing body measurements due to its simplicity, but it cannot differentiate between fat and muscle. WC is proposed as a complementary metric that is particularly effective for detecting central obesity. However, excluding height in the WC measurement may result in inaccuracies when estimating abdominal obesity across individuals of varying statures, thereby underscoring its limitations (5). Thus, we included WHtR analysis. In the fully adjusted model, a one-hour increase in sleep time was associated with a 0.19 kg/m^2^ decrease in BMI (*β* = −0.19, 95%CI: −0.26, −0.12). After full adjustment, each additional hour of sleep was associated with a 0.36 cm decrease in WC (β = −0.36, 95%CI: −0.53, −0.19). After full adjustment, a one-hour increase in sleep duration was associated with a 0.27% decrease in WHtR expressed as a percentage (β = −0.27, 95%CI: −0.38, −0.17). The subgroup analysis results suggested that statistically significant differences were observed regarding race, diabetes, or hypertension status. Thus, the above findings apply more to middle-aged and elderly Non-Hispanic White in the U.S. However, we found that the number of participants in specific races was much lower than that in other subgroups, which may decrease the power of significant effect detection.

Previous studies, including meta-analyses and systematic reviews, have consistently shown a correlation between sleep duration and obesity indicators (14–27, 39–41). According to the Korean Health and Nutrition Survey study conducted by Kim et al. (28), those who slept less than 5 h per day had an increased likelihood of obesity, as determined by their BMI. In a large number of wearable sensor participants, the higher the body mass index, the shorter the sleep duration (29). Another study by Yazdanpanah et al. (41) found that BMI, WC and WHtR were in a significant negative association with nighttime sleep in an Iranian population. Furthermore, a meta-analysis conducted by Bacaro et al. (30) discovered that adults with a shorter sleep duration were more prone to a heightened susceptibility to obesity, but results on long sleep were still inconclusive. These studies reveal a negative correlation between sleep duration and obesity indicators in the case of short sleep duration, although the effects of long sleep remain uncertain. Our results are consistent with theirs on short sleep conditions. At the same time, we found that in the case of appropriate and long sleep, sleep duration and BMI, WC still showed a negative correlation, which is a new finding. The shape of the curve suggested that the critical value of sleeping is 7 h (even though this cut-off value was not significant) and that sleeping less than 7 h can be more associated with worse anthropometric indicators. In a comparable cross-sectional study, the associations between sleep duration and obesity indicators were more pronounced in young adults than in older adults (40), so studies tend to target younger groups. Our article, which focuses on the middle-aged and elderly population in the United States, strengthens the research analysis on older adults.

Multiple mechanisms elucidate the associations between sleep duration and obesity indicators (42). Several researches have shown that during sleep the secretion of various hormones varies, contributing to the metabolism and energy balance of our body (43–47). Cortisol secretion has a pronounced circadian rhythm‌, reaching a trough at midnight (48). However, sleep deprivation disrupts the hypothalamic–pituitary–adrenal (HPA) axis and the circadian rhythm of cortisol, increasing cortisol levels and promoting increased fat storage (43–45). Thyroid hormones regulate basal metabolic rate and energy expenditure (49). Short sleep duration disrupts thyroid function, reducing its levels and slowing metabolism, leading to an energy surplus and weight gain (47). Additionally, short sleep duration decreases leptin levels and increases ghrelin, enhancing appetite (46). Additionally, people with short sleep duration may increase eating possibilities because of the longer period of wakefulness (50, 51). Some studies have found no significant relationship between sleep duration and energy consumption (47, 52, 53). However, other studies have shown that shorter sleep times increase their energy expenditure, but at the same time, their energy intake may be higher (54–57). Many other researchers have also investigated the link between long sleep and obesity, but there are limited mechanisms to explain this relationship (58).

Our study has several strengths. First, an extensive dataset obtained from NHANES produced a representative sample of middle-aged and elderly Americans, to which we can generalise our findings to the broader U.S. population. Second, the confounding covariates were adjusted to make the results more reliable. Third, more detailed statistical analyses were employed, including multiple logistic regression models with corresponding adjustment ranges, smooth curve fitting, and subgroup analysis, to fully assess the correlation between sleep duration and obesity indicators. However, our research still has some shortcomings. Due to the limitations of cross-sectional studies, there is a correlation between the duration of sleep and obesity indicators without causation. In addition, most of the published studies, including ours, relied on self-reported sleep measures (i.e., memory and subjective estimations, which can be imprecise and inaccurate). Subsequent studies could consider introducing other sleep indicators, such as the Pittsburgh Sleep Quality Index, to evaluate sleep quality better (59). Finally, the use of weighting in regression analysis made the conclusions more relevant to the US population but less relevant to the general population.

## Conclusion

Sleep duration was significantly negatively associated with BMI and WC, suggesting that longer sleep duration may contribute to lower obesity indicators in middle-aged and elderly Americans. Subgroup analysis showed that their negative correlation differed between races or hypertension status. However, additional prospective studies are required to validate these findings and investigate potential causal relationships. These findings may facilitate exploring novel weight loss strategies tailored to more specific demographic groups.

## Data Availability

The data presented in the study are deposited in the National Center for Health Statistics(NHANES)repository, accession number NHANES 2005-2014. Link: https://wwwn.cdc.gov/nchs/nhanes/Default.aspx.

## Footnotes

^1^ https://www.cdc.gov/nchs/nhanes
